# miR-23a/b regulates the balance between osteoblast and adipocyte differentiation in bone marrow mesenchymal stem cells

**DOI:** 10.1038/boneres.2016.22

**Published:** 2016-08-24

**Authors:** Qi Guo, Yusi Chen, Lijuan Guo, Tiejian Jiang, Zhangyuan Lin

**Affiliations:** 1 Department of Endocrinology, The Xiangya Hospital of Central South University, Changsha 410008, China; 2 Department of Gerontology, The Xiangya Hospital of Central South University, Changsha 410008, China; 3 Department of Orthopedics, The Xiangya Hospital of Central South University, Changsha 410008, China

## Abstract

Age-related osteoporosis is associated with the reduced capacity of bone marrow mesenchymal stem cells (BMSCs) to differentiate into osteoblasts instead of adipocytes. However, the molecular mechanisms that decide the fate of BMSCs remain unclear. In our study, microRNA-23a, and microRNA-23b (miR-23a/b) were found to be markedly downregulated in BMSCs of aged mice and humans. The overexpression of miR-23a/b in BMSCs promoted osteogenic differentiation, whereas the inhibition of miR-23a/b increased adipogenic differentiation. Transmembrane protein 64 (Tmem64), which has expression levels inversely related to those of miR-23a/b in aged and young mice, was identified as a major target of miR-23a/b during BMSC differentiation. In conclusion, our study suggests that miR-23a/b has a critical role in the regulation of mesenchymal lineage differentiation through the suppression of Tmem64.

## Introduction

Osteoblast-mediated bone formation, a key determinant of bone mass, maintains bone homeostasis along with osteoclast-mediated bone resorption. Bone marrow mesenchymal stem cells (BMSCs), the progenitor cells for osteoblasts, adipocytes, and chondrocytes, show a decrease with age in their potential to differentiate into osteoblasts rather than adipocytes, which results in age-related bone loss and fat accumulation in the bone marrow.^1–4^ Age-related osteoporosis, coupled with an increase in bone marrow fat, is attributable to an imbalance between osteoblast and adipocyte differentiation.^3^ However, the molecular mechanisms that regulate the fate of BMSCs remain unclear.

MicroRNAs (miRNAs) are small (22–24 nucleotides), single-stranded noncoding RNAs that are involved in diverse biological processes. Several miRNAs have been characterized to participate in osteogenesis or adipogenesis. Li *et al.*^5^ found that miR-188 was highly expressed in aged mice and humans and that it regulates the bifurcation of differentiating BMSCs into osteoblasts and adipocytes.^5^ miR-204 and miR-211 have been reported to act as vital negative regulators of Runx2 to promote adipogenesis and suppress osteogenesis in BMSCs.^6^ miR-205 has been shown to exert negative effects on the osteogenic differentiation of BMSCs,^7^ whereas miR-21 promoted the osteogenic differentiation of MSCs via the PI3K/β-catenin pathway.^8^ Despite these findings, the functions of miRNAs in the differentiation and lineage commitment of BMSCs requires further investigation.

In this study, we identified two novel miRNAs, miR-23a, and miR-23b, that are downregulated in the BMSCs of aged vs young mice and humans. We also investigated the roles of these two miRNAs in the differentiation of BMSCs *in vitro*. We demonstrate that miR-23a/b strikingly enhanced osteoblast and attenuated adipocyte differentiation from BMSCs by targeting *Tmem64*. Consequently, our study suggests that miR-23a/b acts as an age-related ‘switch’ to divert BMSCs from being adipogenic to osteogenic.

## Materials and methods

### BMSC culture and transfection

Following the isolation of mouse and human BMSCs, the cells were cultured to the third-passage as previously described.^5^ The miRNA blocker antagomiR-23a/b, analog agomiR-23a/b and their negative control were synthesized by Ribobio (Guangzhou, China). miRNA blockers and analogs were prepared and directly mixed with cells according to the manufacturer’s instructions.

### Clinical samples

Human bone marrow samples were collected from 32 aged patients (17 males and 15 female) who were >70 years old and from 29 young patients (15 males and 14 female) aged from 20 to 40 years old who underwent routine therapeutic surgery at the Orthopedic Surgery Department of the Xiangya Hospital of Central South University.

### Adipogenic differentiation assay and Oil Red staining

To induce adipogenic differentiation, BMSCs were grown in 6-well plates at 2.5×10^6^ cells per well in adipogenic-inducing medium α-MEM (Gibco, Waltham, MA, USA) containing 10% FBS (Gibco), 5 μg·mL^−1^ insulin, 0.5 mmol·L^−1^ 3-isobutyl-1-methylxanthine, and 1 μmol·L^−1^ dexamethasone for 14 days. Culture medium was changed every 2 or 3 days. After 14 days of adipogenic induction, Oil Red O staining was performed to detect the lipid droplets as previously described.^9^

### Osteogenic differentiation and Alizarin Red staining

To induce osteogenic differentiation, BMSCs were grown in 24-well plates at 5×10^5^ cells per well in osteogenic-inducing medium (300 ng·mL^−1^ BMP-2, 50 μg·mL^−1^ ascorbic acid, and 5 mmol·L^−1^ β-glycerolphosphate) for 48 h. An alkaline phosphatase (ALP) activity assay and the assessment of secreted osteocalcin levels were performed using an enzymatic colorimetric ALP kit (Roche Diagnostics, Minneapolis, MN, USA) and a specific immunoassay kit (DiaSorin, Stillwater, MN, USA), respectively, as previously described.^5^ To induce osteoblastic mineralization, BMSCs were grown in mineralization-inducing medium as described above in 6-well plates at 2.5×10^6^ cells per well for 21 days. Alizarin Red staining was performed as previously described.^5^ Spectrophotometry was used to quantify Alizarin Red S at 540 nm.

### qRT-PCR analysis

Quantitative reverse transcription PCR (qRT-PCR) was performed using a Roche Molecular Light Cycler (Basel, Basel-Stadt, Switzerland) as previously described.^10–12^ We used TRIzol reagent (Invitrogen, Carlsbad, CA, USA) to isolate total RNA from cultured cells or tissues, and 1.0 μg total RNA and SuperScript II (Invitrogen) were used to perform reverse transcription. The amplification reactions, which contained amplification primers and SYBR Green PCR Master Mix (Perkin-Elmer Corporation, Applied Biosystems, Foster City, CA, USA), were set up in 25-μL reaction volumes, and a 1 μL of complementary DNA was added to each amplification reaction. The nucleotide sequences of primers for *β-actin*, *Tmem64*, *Pparg*, *Fabp4*, *Runx2*, *Osterix*, miR-23a/b, and U6 are listed in Tables 1 and 2.

### Western blot

Western blotting was performed as previously described.^13–15^ Total cell lysates were separated by SDS-polyacrylamide gel electrophoresis and then transferred to PVDF membranes (Millipore, Bedford, MA, USA). Tmem64 levels were detected using an anti-Tmem64 antibody (sc-87460; Santa Cruz, Dallas, TX, USA) and were normalized to β-actin (ab3280; Abcam, Cambridge, MA, USA). The membranes were incubated with appropriate HRP-conjugated secondary antibodies, and the blots were visualized using an ECL kit (Santa Cruz) and exposed to X-ray films.

### mRNA 3′-UTR cloning and luciferase reporter assay

A segment of the mouse *Tmem64* 3′-untranslated region (UTR) containing the predicted miR-23a/b binding site was amplified by PCR using the forward primer 5′- CTAGAGGAATTCTGAAATGTGAAATTGTCTCAAGGCCGG - 3′ and the reverse primer 5′- CCTTGAGACAATTTCACATTTCAGAATTCCT-3′. The PCR products were purified and inserted into the *Xba*I–*Fse*I site downstream of the stop codon in the pGL3 control luciferase reporter vector (Promega, Madison, WI, USA), resulting in WT-pGL3-*Tmem64*. A QuikChange site-directed mutagenesis kit (Stratagene, La Jolla, CA, USA) was used to insert mutations into the miR-23a/b seed region to obtain MUT-pGL3-*Tmem64*. The primers for *Tmem64* 3′-UTR mutagenesis were 5′- CTAGAGGAATTCTGAACACTGAAATTGTCTCAAGGCCGG-3′ (forward) and 5′- CCTTGAGACAATTTCAGTGTTCAGAATTCCT-3′ (reverse).

BMSCs were transfected with wild type (WT) or MUT-pGL3-*Tmem64* constructs (200 ng) and either agomiR-23a/b or agomiR-NC for 48 h using Lipofectamine 2000 (Invitrogen) according to the manufacturer’s instructions. The modified pGL3 control vector without a 3′-UTR insert was used as a positive control. BMSCs treated with Lipofectamine only served as negative controls. The luminescence signal was quantified by a dual luciferase reporter assay system (Promega) using a luminometer (Glomax, Promega). Values from the firefly luciferase assay were normalized to the Renilla luciferase assay value from the transfected phRL-null vector (Promega).

The 3′-UTR of mouse *Tmem64* was amplified by RT-PCR from total RNA extracted from BMSCs and using primers designed based on the mouse *Tmem64* complementary DNA sequence. The forward primer was 5′- GCTCTAGATTGTTGAGAGCCTAGCGTGC-3′, and the reverse primer was 5′- GCGGTACCCAGCTCAGACGTACCAGGTC-3′. A QuikChange site-directed mutagenesis kit (Stratagene) was used to generate the two mutations in *Tmem64* (mutant *Tmem64*) by PCR using the WT *Tmem64* construct as the template. The introduced mutations did not result in amino-acid changes in the Tmem64 protein. Finally, WT and mutant *Tmem64* were cloned into the pCDNA3.1 (+) expression vector (Invitrogen) at *Xba*I/*Kpn*I sites. Then, we co-transfected the WT or the mutant *Tmem64* 3′-UTR construct with agomiR-23a/b into mouse BMSCs as described above.

### Statistical analyses

Statistics were analyzed using SPSS 16.0 (Polar Engineering and Consulting, http://www.winwrap.com/). Data are presented as the mean±s.d. A Student’s *t*-test was used for comparing the differences between two groups. Comparisons of multiple groups were made using one-way ANOVA. All experiments were repeated at least three times, and representative experiments are shown. *P*<0.05 was considered statistically significant.

### Study approval

All animal care protocols and experiments were reviewed and approved by the Animal Care and Use Committee of the Laboratory Animal Research Center at Xiangya Medical School of Central South University. All female WT C57BL/6 mice of different ages used in experiments were housed under specific pathogen-free conditions (22 °C, 12-h light/12-h dark cycles, and 50%–55% humidity) with free access to food pellets and tap water.

The clinical study was approved by the Ethics Committee of Central South University, and informed consent was obtained from each participant before the collection of clinical samples.

## Results

### miR-23a/b is markedly downregulated in BMSCs during the aging process

**Figure 1:**
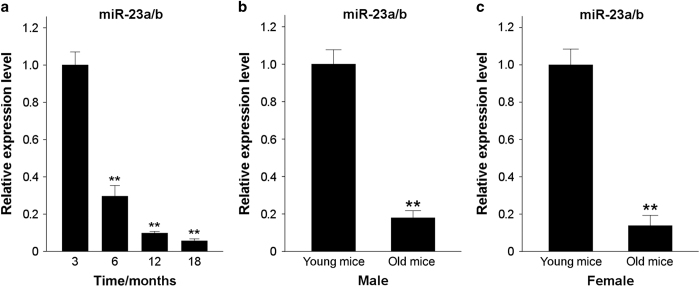
miR-23a/b is gradually downregulated in BMSCs throughout the aging process. (**a**) qRT-PCR was used to analyze the relative levels of miR-23a/b in BMSCs isolated from C57BL/6 mice of different ages. *n*=5 per group. (**b** and **c**) Comparison of miR-23a/b levels in young and old human BMSCs as determined by qRT-PCR of human male (**b**) and female samples (**c**). Male: *n*_y_=15, *n*_o_=17. Female: *n*_y_=14, *n*_o_=15. Data are shown as the mean±s.d. **P<0.01 (ANOVA or Student’s *t*-test). ANOVA, analysis of variance; BMSCs, bone marrow mesenchymal stem cells; qRT-PCR, quantitative reverse transcription PCR.

Our previous findings^5^ revealed that BMSCs tended to differentiate into adipocytes rather than osteoblasts over the course of aging. To investigate the differences in the miRNA expression profiles of BMSCs from young and aged mice, we had previously performed microarray analysis to identify dysregulated miRNAs.^5^ We identified miR-23a/b to be the most significantly downregulated miRNAs in aged vs young mice, and in this study, we chose to study miR-23a/b further and investigated its function in the regulation of BMSC differentiation. We confirmed the decreased level of miR-23a/b qRT-PCR (Figure 1a), and we then measured the levels of miR-23a/b in human BMSCs that were isolated from the bone marrow cells of patients aged either 20 to 40 or >70 years. Consistently, the expression of miR-23a/b was notably decreased in elderly samples compared with that in young samples (Figure 1b and c). This result suggests that miR-23a/b is involved in age-related effects on BMSCs in mouse and human.

### miR-23a/b inhibits the adipogenic differentiation of BMSCs

**Figure 2:**
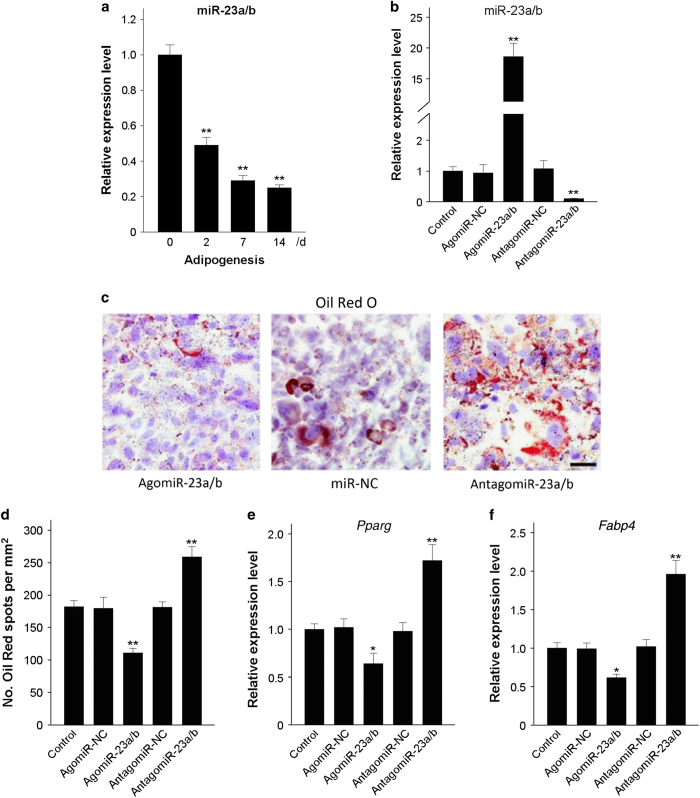
miR-23a/b inhibits the adipogenic differentiation of BMSCs. (**a**) qRT-PCR analysis of the relative levels of miR-23a/b in BMSCs induced to differentiate into adipocytes for 14 days. (**b**) The relative levels of miR-23a/b in BMSCs transfected with 10 μmol·L^−1^ agomiR-23a/b, antagomiR-23a/b or their NC were analyzed by qRT-PCR. (**c** and **d**) Representative images of Oil Red staining of lipid droplets (**c**), and the quantitative analysis of the number of Oil Red spots (**d**) in BMSCs induced to differentiate into adipocytes for 14 days. (**e** and **f**) The relative mRNA expression levels of adipogenic markers, Pparg (**e**) and Fabp4 (**f**), were measured by qRT-PCR in BMSCs induced to differentiate into adipocytes for 48 h. Scale bars: 120 μm. *n*=5 per group. Data are shown as the mean±s.d. *P<0.05, **P<0.01 (ANOVA). ANOVA, analysis of variance; BMSCs, bone marrow mesenchymal stem cells; NC, negative controls; qRT-PCR, quantitative reverse transcription PCR.

miR-23a/b expression was revealed by qRT-PCR analysis to gradually decrease during adipogenic differentiation in the BMSCs of 6- to 8-week-old mice (Figure 2a). To overexpress or silence miR-23a/b in BMSCs for functional investigation, we transfected BMSCs with agomiR-23a/b, antagomiR-23a/b or their negative control and subsequently induced adipogenic differentiation (Figure 2b). The overexpression of miR-23a/b attenuated lipid droplet formation in adipogenesis-induced BMSCs (Figure 2c and d). Likewise, the messenger RNA (mRNA) levels of two important adipocyte markers, peroxisome proliferator-activated receptor-g (Pparg) and fatty acid binding protein 4 (Fabp4), were also reduced compared with controls (Figure 2e and f). Conversely, silencing miR-23a/b promoted lipid droplet formation (Figure 2c and d) and increased the levels of *Pparg* and *Fabp4* mRNA during the adipogenic differentiation of BMSCs (Figure 2e and f). Taken together, these observations suggest that miR-23a/b negatively regulates the adipogenic differentiation of BMSCs.

### miR-23a/b promotes the osteogenic differentiation of BMSCs

**Figure 3:**
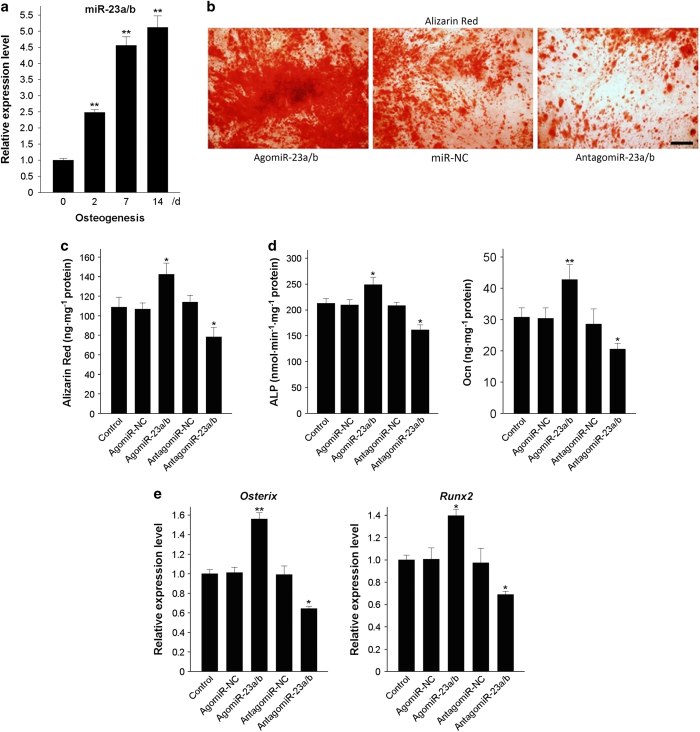
miR-23a/b promotes the osteogenic differentiation of BMSCs. (**a**) qRT-PCR analysis showed the relative levels of miR-23a/b in BMSCs induced to differentiate into osteoblasts for 14 days. (**b** and **c**) Representative images of Alizarin Red staining (**b**) and the quantitative analysis of matrix mineralization (**c**) in BMSCs induced to differentiate into osteoblasts for 21 days after transfection. (**d**) ALP activity and osteocalcin secretion were measured in BMSCs induced to generate osteoblasts for 48 h. (**e**) qRT-PCR was used to analyze the relative expression levels of *Osterix* and *Runx2* in BMSCs induced to differentiate into osteoblasts for 48 h. Scale bars: 100 μm. *n*=5 per group. Data are shown as the mean±s.d. *P<0.05, **P<0.01 (ANOVA). ANOVA, analysis of variance; BMSCs, bone marrow mesenchymal stem cells; qRT-PCR, quantitative reverse transcription PCR.

Our results showed that miR-23a/b expression gradually increased in BMSCs from 6- to 8-week-old mice during the process of osteoblastic differentiation (Figure 3a). We next determined the role of miR-23a/b during the osteogenic differentiation of BMSCs by overexpressing or silencing miR-23a/b in BMSCs. After being transfected with agomiR-23a/b, antagomiR-23a/b, or their negative controls, BMSCs were cultured in osteogenic-inducing medium. Alizarin Red staining indicated that the overexpression of miR-23a/b facilitated the osteogenic differentiation of BMSCs, whereas the silencing of miR-23a/b inhibited osteogenic differentiation (Figure 3b and c).

In addition, ALP activity and osteocalcin secretion, both markers of osteoblast differentiation, were evaluated in agomiR-23a/b-transfected BMSCs and compared with control BMSCs (Figure 3d). Furthermore, the mRNA levels of two critical osteoblast transcription factors, Osterix, and Runx2, were also increased following transfection with agomiR-23a/b (Figure 3e). In contrast, the transfection of antagomiR-23a/b attenuated ALP activity and osteocalcin secretion, in addition to Osterix and Runx2 expression (Figure 3d and e). Altogether, all of these data indicate that miR-23a/b enhances the osteogenic differentiation of BMSCs.

### miR-23a/b directly targets *Tmem64*

miRNAs have been shown to regulate the expression of mRNAs by binding to coding sequences or the 3’-untranslated regions (3′-UTRs) of target genes.^16^ We used Starbase v2.0 (http://starbase.sysu.edu.cn/contact.php/; Li *et al.* Nucleic Acids Res. 2014 & Yang *et al.* Nucleic Acids Res. 2011) to predict the possible target genes of miR-23a/b, considering the predicted intersections of miRanda, PicTar, and TargetScan and using medium stringency. Among the 26 possible target genes predicted, we chose Transmembrane protein 64 (Tmem64), which had been reported to participate in the regulation of mesenchymal lineage allocation,^9^ for further analysis.

**Figure 4:**
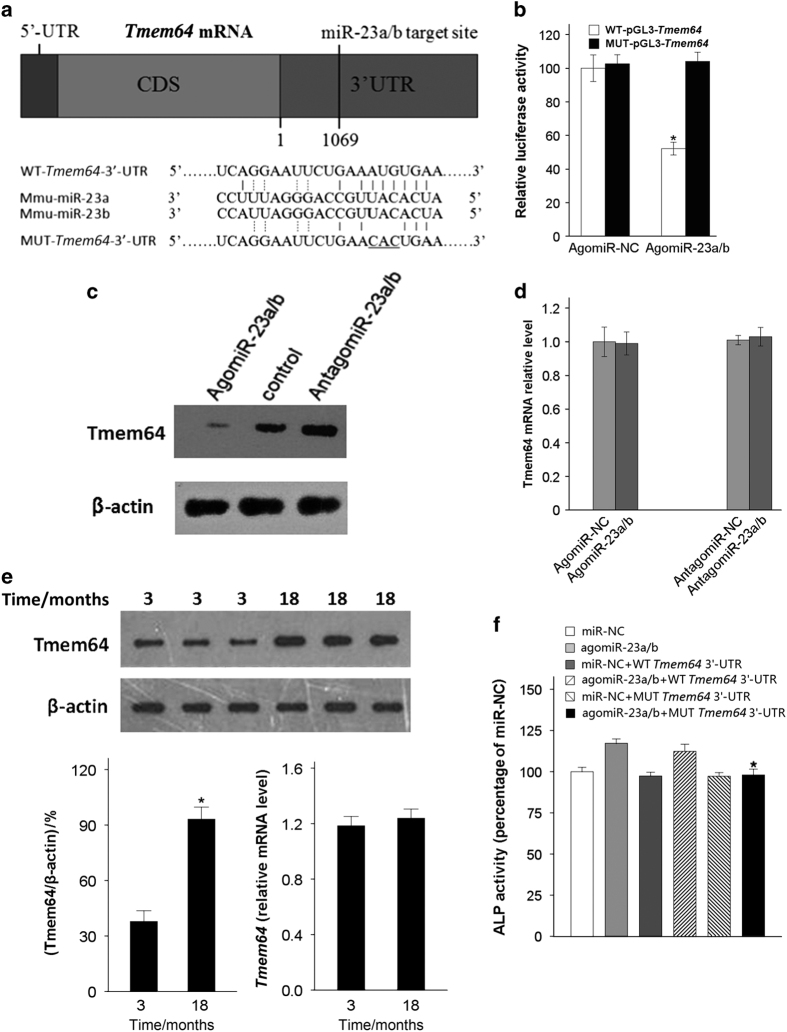
miR-23a/b directly targets *Tmem64*. (**a**) Schematic representation of the predicted miR-23a/b target site in the 3′-UTR of mouse *Tmem64*. The alignment of miR-23a/b with WT and MUT 3′-UTR region is shown by complementary pairing, and three mutated nucleotides are underlined. (**b**) BMSCs were co-transfected with the luciferase reporter carrying WT-pGL3-*Tmem64* or MUT-pGL3-*Tmem64* along with agomiR-23a/b or agomiR-NC. The effects of miR-23a/b on the luciferase reporter constructs were determined 48 h after transfection. The firefly luciferase values were normalized to Renilla luciferase; *n*=5. (**c**) After BMSCs were transfected with agomiR-23a/b or antagomiR-23a/b, the relative levels of Tmem64 protein expression were determined by western blot; β-actin was used as loading control; *n*=5. (**d**) The relative levels of *Tmem64* mRNA were determined using qRT-PCR and normalized to β-actin; *n*=5. (**e**) Tmem64 protein levels in BMSCs from 3- and 18-month-old mice were measured by western blot and expressed as the densitometry of Tmem64/β-actin. *Tmem64* mRNA levels were determined by qRT-PCR and are shown as the fold-induction relative to β-actin; *n*=3. (**f**) The increase in ALP activity induced by agomiR-23a/b was blocked by the transfection of MUT *Tmem64* 3′-UTR into osteogenic-induced-BMSCs. *P<0.05 vs. agomiR-23a/b+WT *Tmem64* 3′-UTR. *n*=3. Data are shown as the mean±s.d. *P<0.05 (Student’s *t*-test or ANOVA). ALP, alkaline phosphatase; ANOVA, analysis of variance; BMSCs, bone marrow mesenchymal stem cells; CDS, coding sequence; qRT-PCR, quantitative reverse transcription PCR; WT, wild type; 3′-UTR, 3′-untranslated region.

Sequence analysis showed one miR-23a/b binding site in the 3′-UTR of the *Tmem64* gene (position 1069-1076; Figure 4a). To clarify whether miR-23a/b could directly target the *Tmem64* gene, a luciferase reporter construct including the putative binding site of the *Tmem64* 3′-UTR (WT-pGL3-*Tmem64*) was generated, and three mutant nucleotides were introduced into the predicted target sequences (MUT-pGL3-*Tmem64*) and used as a control. We transfected WT-pGL3-*Tmem64* or MUT-pGL3-*Tmem64* along with agomiR-23a/b or agomiR-NC into BMSCs and assessed the effects of miR-23a/b on luciferase translation by luciferase enzyme activity. Transfection with agomiR-23a/b was able to repress the luciferase activity of the *Tmem64* 3′-UTR reporter gene, whereas MUT-pGL3-*Tmem64* prevented this inhibition (Figure 4b). This finding confirmed that miR-23a/b can specifically bind to the predicted 3′-UTR of *Tmem64*.

To further determine whether this conserved site was the actual binding region, we transfected BMSCs with agomiR-23a/b or antagomiR-23a/b and detected the mRNA and protein levels of Tmem64. The overexpression of miR-23a/b decreased endogenous levels of Tmem64 protein, whereas the inhibition of miR-23a/b elevated Tmem64 protein levels (Figure 4c); however, *Tmem64* mRNA levels remained stable (Figure 4d). We also measured the levels of Tmem64 mRNA and protein in BMSCs from of 3- and 18-month-old mice, and we found increased levels of Tmem64 protein in aged mice; however, the increase of *Tmem64* mRNA in aged mice was not statistically significant (Figure 4e).

We next co-transfected the WT or mutant *Tmem64* 3′-UTR construct along with agomiR-23a/b or miR-NC into mouse BMSCs cultured in osteogenic-inducing medium; miR-NC and agomiR-23a/b were used as negative and positive controls, respectively. We observed an increase in ALP activity in those cells transfected with agomiR-23a/b and agomiR-23a/b+WT *Tmem64* 3′-UTR. The increase in ALP activity induced by agomiR-23a/b was blocked by the mutant *Tmem64* 3′-UTR construct (Figure 4f). These results together show that *Tmem64* shows increased expression with age and is the major target of miR-23a/b during BMSC differentiation and that miR-23a/b affects Tmem64 expression at the post-transcriptional level.

## Discussion

The maintenance of bone homeostasis primarily depends on osteoblast-mediated bone formation and osteoclast-mediated bone resorption. During the aging process, BMSCs show a gradual decline in their capacity to differentiate into osteoblasts vs adipocytes, which results in progressive bone loss and fat accumulation and leads to age-related osteoporosis.^3,17–22^ However, the mechanism behind this switch in differentiation potential requires further investigation.

In the present study, we observed that miR-23a/b is prominently downregulated in BMSCs of aged mice and humans. The overexpression of miR-23a/b promoted the osteogenic differentiation of BMSCs, whereas the inhibition of miR-23a/b intensified adipogenic differentiation from BMSCs *in vitro*. Furthermore, we determined that miR-23a/b regulated BMSCs differentiation by directly targeting *Teme64*. These results suggest that miR-23a/b has a critical role in BMSC differentiation.

Previously, we performed miRNA microarray analysis to determine that miR-188 becomes remarkably elevated in BMSCs with age, and we identified its vital function in determining the differentiation potential of BMSCs. However, the regulation of BMSC differentiation involves multiple miRNAs. To further investigate the age-related switch in differentiation potential of BMSCs, we observed and identified two important downregulated miRNAs, miR-23a and miR-23b, in the BMSCs of aged mice. In addition, we confirmed that the level of miR-23a/b expression in human BMSCs also showed significant age-related differences. Taken together, these findings indicate that miR-23a/b has a crucial effect on the aging process of BMSCs in both mouse and human.

miR-23a and miR-23b belong to the same family and have strong similarities in their nucleotide sequences, and importantly, they function as synergistic regulators of BMSC functions. Previously, it had been reported that miR-23a/b reinforces the expression of glutaminase in mitochondria and participates in glutamine metabolism.^23^ Several studies have shown that the activation of miR-23a by NFATc3 regulates cardiac hypertrophy^24^ and that miR-23b inhibits autoimmune inflammation.^25^ However, there had been no studies of the action of miR-23a/b on the regulation of BMSC differentiation.

Our study confirmed that miR-23a/b has promoting effects on the osteogenic differentiation of mouse BMSCs *in vitro*. However, Hassan and colleagues have reported that miR-23a had an inhibitory role in the maturation of primary rat osteoblasts and mouse MC3T3-E1 cells through the targeting of SATB2.^26^ These two results are not likely to be contradictory because different cell types show specificity and different mechanisms of action, which could explain these inconsistent results. Moreover, a microRNA can regulate the expression of multiple target genes; therefore, the miR-23 target genes that are relevant to osteoblast maturation and BMSC differentiation might be different. In the present study, we demonstrated that *Tmem64* was the major target of miR-23a/b during mouse BMSC differentiation.

miRNAs have been reported to downregulate gene expression by inhibiting mRNA translation or reducing mRNA stability through binding to sites in the CDS or 3′-UTR of the target gene.^27^ Studies have indicated that *Fas*,^28^ *Runx2*,^29^ *CXCL12*,^29^ *Has2*,^30^ and *Hes1* (ref. 31) are potential target genes of miR-23a or miR-23b. In this study, we demonstrated that *Tmem64* was directly targeted by miR-23a/b and was responsible for regulating BMSC differentiation. Tmem64 has been found to positively modulate osteoclast differentiation via RANKL-mediated Ca^2+^ signaling pathway.^32^ Recently, it was shown that mice in which the *Tmem64* gene was silenced presented increased osteoblast and decreased adipocyte differentiation from BMSCs. Conversely, the overexpression of Tmem64 accelerated adipogenesis and inhibited osteogenesis. Tmem64 regulates the switch in the lineage commitment of MSCs to adipogenesis rather than to osteogenesis by suppressing β-catenin, the key Wnt signaling molecule.^9^ Our study revealed that miR-23a/b mediates BMSC differentiation by post-transcriptionally repressing Tmem64 expression. The decline in miR-23a/b expression in BMSCs with age results in an attenuation of the suppression of Tmem64 and consequently the increased expression of Tmem64 protein, which inhibits the Wnt/β-catenin signaling pathway. Consequently, the regulation of Tmem64 causes BMSCs to have a tendency towards favoring differentiation into adipocytes rather than osteoblasts.

A previous study^5^ revealed that the level of miR-188 expression is markedly higher in BMSCs from aged compared with young mice and humans, and the BMSC-specific inhibition of miR-188 stimulated new bone formation. In the present study, our results showed that miR-23a/b levels are decreased in BMSCs from aged compared with young mice and humans, and the activation of miR-23a/b in BMSCs promoted osteogenic differentiation. These findings suggest that the upregulation of miR-23a/b in BMSCs could be a potential therapeutic target for osteoporosis.

## Figures and Tables

**Table 1:** Primer sequences used for qRT-PCR detection for mRNAs

Gene	Acc. No	Primer sequence (5′ to 3′)
*Pparg* (mouse)	NM_001127330	F: GACCACTCGCATTCCTTT
		R: CCACAGACTCGGCACTCA
*Fabp4* (mouse)	NM_024406	F: AAATCACCGCAGACGACA
		R: CACATTCCACCACCAGCT
*Runx2* (mouse)	NM_001146038	F: ACTTCCTGTGCTCCGTGCTG
		R: TCGTTGAACCTGGCTACTTGG
*Osterix* (mouse)	NM_130458	F: ACCAGGTCCAGGCAACAC
		R: GCAAAGTCAGATGGGTAAGTAG
*Tmem64* (mouse)	NM_181401	F: AGGAAGCGGCCTGAAGGT
		R: GAAGGAAGAGCCACTGGGAT
*β-actin* (mouse)	NM_007393	F: CTGTCCCTGTATGCCTCTG
		R: TGATGTCACGCACGATTT

Acc. No, Genbank accession numbers; F, forward primer; mRNA, messenger RNA; R, reverse primer; qRT-PCR, quantitative reverse transcription PCR.

**Table 2:** Primer sequences used for qRT-PCR detection for microRNA

microRNA	Primer	Primer sequence (5′ to 3′)
miR-23a	RT primer	GTCGTATCCAGTGCAGGGTCCGAGGTATTCGCACTGGATACGAC GGTAATC
miR-23b	Forward	GAGTGATCACATTGCCAGG
	Reverese	GCAGGGTCCGAGGTATTC
U6	RT primer	GAACGCTTCACGAATTTGCGTGTCAT
	Forward	CTCGCTTCGGCAGCACA
	Reverese	AACGCTTCACGAATTTGCGT

qRT-PCR, quantitative reverse transcription PCR.
